# Biomarkers in psychiatry: drawbacks and potential for misuse

**DOI:** 10.1186/1755-7682-3-1

**Published:** 2010-01-12

**Authors:** Shaheen E Lakhan, Karen Vieira, Elissa Hamlat

**Affiliations:** 1 Global Neuroscience Initiative Foundation, Los Angeles, CA, USA

## Abstract

For more than 20 years, researchers have attempted to identify diagnostic and prognostic biomarkers for psychiatric disorders including schizophrenia, major (unipolar) depression, and bipolar disorder. Advocates of this research contend that identifying such biomarkers will aid in the diagnosis of these disorders, as well as the possible development of effective psychiatric medications to treat them. Currently, there are no diagnostic tests available. This is largely due to the multi-factorial nature of psychiatric disorders. Biomarker testing of individuals is also prohibitively expensive because significant expertise is required to conduct tests and follow-up counseling for the patient is often necessary. It is cautioned that widespread biomarker testing could lead to negative consequences such as discrimination in health insurance and employment, as well as selective abortion.

## Correspondence

There are no clinical laboratory tests to date that can be used by clinicians to diagnose patients with psychiatric disorders. Instead, psychiatrists have to rely on the patient's description of symptoms, mental status examinations, and clinical behavioral observations in order to make an accurate diagnosis in line with the diagnostic categories listed in the Diagnostic and Statistical Manual of Mental Disorders 4th Edition (DSM-IV) [1] or the International Statistical Classification of Diseases and Related Health Problems 10^th ^Revision (ICD-10) [2]. Unfortunately, it can take months or even years for clinicians to make a correct psychiatric diagnosis using current methods and there is much room for error. Quantitative criteria are lacking for specific psychiatric disorders, and many diagnostic criteria overlap.

It has been widely accepted that etiological diagnosis of medical illness is superior to syndromal (symptom-based) diagnosis. Scientific investigations into biomarkers for schizophrenia, major depression, and bipolar disorder have the goal of generating more definitive diagnostic tools for these disorders. The multidisciplinary approach of Convergent Functional Genomics [3] has revealed multiple candidates. This approach integrates animal model gene expression data with human genetic linkage/association data, as well as human tissue (postmortem brain, blood) data, to cross-validating findings, extract meaning from large datasets, and prioritize candidate genes, pathways, and mechanisms for subsequent hypothesis-driven research [4]. These potential biomarkers can be divided into three primary categories: protein-based, imaging-linked, and genetic (see [5] for a recent review).

Easily accessible bodily fluids like blood, urine, and cerebral spinal fluid (CSF) are potential sources for the identification of protein-based psychiatric biomarkers. CSF, however, is probably the most relevant source for identifying protein biomarkers for patients with psychiatric disorders because proteins that have been secreted or shed from brain cells can be found there. These proteins also can be found in the blood due to an exchange with CSF; however, their levels are much reduced. As a result, researchers are focusing on CSF as a means for biomarker discover and blood for an eventual clinical diagnostic assay.

Huang et al. utilized surface-enhanced laser desorption ionization mass spectrometry in a total of 179 cerebrospinal fluid samples (58 schizophrenia patients, 16 patients with depression, 5 patients with obsessive-compulsive disorder, 10 patients with Alzheimer disease, and 90 controls). They discovered three key schizophrenia-specific alterations in the up-regulation of a 40-amino acid VGF-derived peptide, the down-regulation of transthyretin at 4 kDa, and a peptide cluster at 6,800-7,300 Da (which is likely to be influenced by the doubly charged ions of the transthyretin protein cluster) [6].

Genetic biomarkers are specific genes, mutations, and single nucleotide polymorphisms (SNPs) that can be linked to psychiatric symptoms. In one study, using blood samples from patients with mood disorders, the investigators were able to determine 11 different biomarker candidates: 5 genes involved in myelination and 6 genes involved in growth factor signaling [7]. There was also prior evidence of the differential expression of these genes in the postmortem brains of individuals suffering from mood disorders.

To be truly useful, a psychiatric biomarker must have predictive power and allow for the identification of at-risk individuals [8]. Proponents of determining clinically useful, cost-effective biomarkers believe it will enhance patient management, improve treatment and therapeutic response, and lead to targeted therapy tailored to the individual [9]. They also point to the possibility of increased positive outcome through early intervention.

Genetic biomarkers are one area in psychiatric disorders that is being researched, but neuroimaging is another research tool being investigated to aid in the diagnosis and etiology of psychiatric illnesses. The general hypothesis is that the neruoanatomy of people afflicted with a psychiatric disorder will differ than those who do not have a psychiatric disorder. One neuroimaging study by Borgwardt et. al investigating schizophrenia in twins demonstrated that monozygotic twins afflicted with schizophrenia had reduced brain gray matter volume versus healthy monozygotic twins [10]. One interesting observation of this study is that they also included monozygotic twins in which one twin was diagnosed with schizophrenia while the other was not (discordant). In the discordant twins the one with schizophrenia had reduced gray matter volume just like the concordant twins; however, the regions of reduced gray matter volume were different. This finding suggests that genetic influence elicits a different etiology than environmental influences on the same outcome of schizophrenia.

General anxiety disorder (GAD) is another psychiatric disorder that affects many people. Just like with schizophrenia, there is extensive research underway to determine the etiology of GAD. Neuroimaging is one tool researches are using to decipher this disorder. Using MRI, researchers found that people afflicted with GAD had different amygdalar connectivity than control subjects [11]. The amygdalae are part of the limbic system and are responsible for developing memories tied to emotional events. MRI is also being used to investigate neuroanatomy differences in people diagnosed with obsessive-compulsive disorder (OCD). One recent study showed that people with OCD had different cortical folding versus control subjects [12]. Neuroimaging to detect differences in neuroanatomy in those with psychiatric disorders is in its infancy, but will likely lead to great advancements in the treatment, diagnosis, and understanding of these disorders.

Another area of intense research is on inflammatory agents that may add to the complexity of psychiatric disorder development. Inflammatory agents such as cytokines or specific hormones have been implicated as markers of psychiatric disorders. This is mainly due to the observation that people afflicted with depression have higher inflammatory markers such as interleukin-6 or cortisol [13-15]. In one study researchers measured the inflammatory response of pregnant women receiving the flu vaccine [13]. As a marker of inflammation researchers used macrophage migration inhibitory factor (MIF), a cytokine up-regulated during inflammation. The flu vaccine was used as an *in vivo *antigen challenge, which all women who are pregnant during flu season should get. The women were separated by depression status. The authors observed that women previously diagnosed with major depressive disorder or bipolar had higher MIF responses than those women with no psychiatric disorder, suggesting a dysregulation of inflammatory responses during depression.

Thorough assessment, however, is needed before we are to completely change the way in which we diagnose and treat patients with psychiatric disorders. Instead of simply focusing on its potential, a closer look at the drawbacks and possible misuses of this new technology is needed.

## Biomarker Drawbacks

### Cost

The first major issue associated with any new technology is almost always its cost; biomarker screening is no exception. Diagnostic testing can be extremely expensive. Genetic testing can cost anywhere from $100 to $3,000 per patient [16]. For example, implementing a screening program for people with a family history of schizophrenia would be a huge cost burden on insurance companies, health maintenance organizations, or the patients themselves. If an individual tests positive for a genetic biomarker, additional funds may be needed for post-screening support and counseling and/or treatments for the disorder.

Currently, implementing such screening programs is not worth the investment since genetic biomarkers are not yet highly predictive of psychiatric disease. In the case of one likely genetic biomarker for schizophrenia (neuregulin 1), the most promising findings to date account for only a 1% increased risk of developing symptoms [8]. Thus, despite the high heritability of schizophrenia, single genes only make a very small contribution to the overall risk of disease [17]. In order to be cost effective, any future clinical diagnostic criteria that includes biomarker screening should also take into consideration a patient's reported symptoms, especially those that interfere with their daily life [1]. This way, only individuals who are considerably afflicted by mental disorders will be treated.

### Unreliability

One of the reasons finding genetic biomarkers is so challenging is that most mental disorders are considered multi-factorial; multiple genes or gene families trigger disease manifestation. Commonly referred to as polygenic inheritance, it is believed that specific "gene sets" or "gene combinations" contribute to the development of symptoms. Although investigations are ongoing, we do not know the identity of many of the genes that play a significant role in the genesis of mental illness or the major pathways in which they participate. The linkage of a few genes or gene products to the wide-variety of psychiatric symptoms cannot reliably be used for the diagnosis of mental disorders [17].

The influence of the environment on psychiatric disorders also needs to be considered. Schizophrenia studies involving monozygotic twins have shown that twins may have the same genotype, but not have the same phenotype [18], with one twin going on to develop schizophrenia while the other does not. Non-genetic factors may play a key role in disease development and progression. Socio-economic statuses, access to technology, and even religious influence have been shown to modify the onset and severity of psychiatric disorders. For example, a lower socio-economic status is associated with an increased risk of mental illness and psychiatric hospitalization [19,20]. Religious belief has been shown to aid recovery in patients with schizophrenia. In one study, increased religious activity was associated with a decrease in symptoms [21]. However, religion can also become part of the patient's problem if they are rejected by their faith community, burdened by spiritual activities, and demoralized by their beliefs [22]. Since those afflicted by psychiatric disorders will not be exposed to the same environmental influences, it is unwise to make generalizations based solely on genetic biomarkers.

The introduction of diagnostic biomarkers can only provide limited information on the *likelihood *of psychiatric disorder development and only on a patient-by-patient basis. Environmental influences and lifestyle choices figure heavily in disease progression and gene products do not function alone but in complex pathways; one gene may influence the behavior of other genes when exposed to additional risk factors [23,24]. Additionally, biomarkers may be a variable as a function of age, gender, ethnicity, and health status of the patient. Researchers have already found that the onset of schizophrenia is influenced by gender. A study conducted by Häfner indicated that schizophrenia tends to affect women 3 to 4 years later than men and women tend to have milder forms of the disease in their younger years. The author attributed this delayed onset to the protective effects of estrogen [25].

Although extensive research is under way, genetic detection, neuroimaging, or inflammatory responses as means of detecting, treating, or diagnosing psychiatric disorders is still in its infancy. While research efforts to find better and more accurate ways for detection are good natured, the implications of these new means of detection is not always well thought out. If a person expresses a certain gene or gene set indicative of a mental health condition, but has no symptoms do they have the condition or not? There is always the question: did an environmental influence cause a change in gene expression that led to a specific disorder? Most psychiatric disorders are not diagnosed in childhood, yet a person is born with their genetic makeup. There is some missing factor not answerable with genetics alone that leads to a specific phenotype. The same argument can be made for neuroimaging or inflammatory responses. Not every person that is diagnosed with a psychiatric disorder will have a different neuroanatomic make up. Not every person with a neuroanatomic difference will have a psychiatric disorder. So, what will be the exception and what will be the rule?

### Ethical Concerns

Perhaps the biggest concern related to prognostic biomarkers, though, is whether testing would actually be of any benefit to asymptomatic individuals. If researchers could develop a blood test for schizophrenia in the next few years, what are the ethical implications for instituting routine testing for "at-risk" individuals when there is currently a lack of preventative therapeutic strategies [26]?

## Potential for Biomarker Misuse

Research into the identification of biomarkers for psychiatric disorders has raised ethical questions, especially concerning the collection and usage of genetic information. Hereditary information is uniquely personal. It can foretell an individual's medical future, divulge personal information about one's parents, siblings and children, and has a history of being used to stigmatize and victimize individuals [27].

### Discrimination

Since the eruption of the HIV/AIDS pandemic there have been numerous debates as to whether people diagnosed with HIV/AIDS should be classified as a protected demographic under non-discriminatory legislation [28]. The discovery of HIV/AIDS was made over two decades ago and despite extensive research and knowledge there is still a stigma attached to those afflicted with the disease. Questions that still arise, ask whether these people should have a right to privacy concerning matters associated with their illness [29,30]. Discrimination is still a problem for this demographic; one study published this year showed that HIV/AIDS sufferers have a harder time finding dental care. Some were refused once they disclosed their illness and the discrimination was more pronounced if the patient was African-American [31].

Another stigmatizing disease is tuberculosis (TB), which was once eradicated from the US but is on the rise again. Recent studies have shown that people's lack of knowledge about TB has driven an overt discrimination against TB sufferers [32,33]. The media has contributed to this stigma by sensationalizing rare cases of antibiotic-resistant TB strains. One study published in 2008 reported that people given a survey asking about their knowledge of TB knew that it was an infectious disease that affected the lungs, but when asked how it was transmitted some thought TB was acquired via sexual transmission [32].

Based on recent studies, there is still a stigma attached to diseases such as HIV/AIDS and TB that have extensive research and knowledge about causative agents, transmission, and treatment. Yet people, including the healthcare field, continue to discriminate against these individuals. If there were a definitive test or biomarker for psychiatric illness, psychiatric disorder patients would likely be exposed to similar discrimination based on historical findings of other stigmatizing disorders. Unfortunately in research, science almost always precedes legislation.

One of the biggest concerns with the collection of genetic or other biological information is that it can be used as an employment and insurance screening tool to deny an otherwise healthy individual employment or healthcare coverage [34]. An actual case of such discrimination involved a mother who had an alpha1-antitrypsin deficiency, an autosomal recessive disorder that can lead to emphysema and liver disease. In 2003, Heidi Williams was denied health insurance for her two children because they were carriers of the disease. This was despite the fact they were both healthy and neither had the two copies of the allele necessary to make them sick [35]. A person is considered to have the disease (AAT deficiency) only if both genes are inherited. Otherwise, people with only one gene are considered 'carriers' -- in this case, AAT levels are lower than normal, but do not cause serious health problems.

In order to combat such prejudice, President Bush signed the Genetic Information Non-discrimination Act (GINA) in 2008, which prevents insurance companies from denying coverage or increasing premium rates to otherwise healthy individuals on the basis of genetic information [36]. Employers are also barred from using genetic information for hiring, firing, or job-placement decisions [37].

However, the scope of this law is limited to "genetic information," which is defined as:

• An individual's genetic test (including genetic tests done as part of a research study);

• Genetic tests of the individual's family members (defined as dependents and up to and including fourth degree relatives);

• Genetic tests of any fetus of an individual or family member who is pregnant, and genetic tests of any embryo legally held by an individual or family member utilizing assisted reproductive technology;

• The manifestation of a disease or disorder in family members (family history);

• Any request for, or receipt of, genetic services or participation in clinical research that includes genetic services (genetic testing, counseling, or education) by an individual or family member.

GINA does not protect the results of tests that do not measure DNA, RNA, or chromosomal changes. Therefore, there is nothing in the law to protect asymptomatic individuals who test positive for non-genetic biomarkers of psychiatric disease, leaving them vulnerable to discrimination.

### Selective Abortion

In the future, parents could conceivably employ prognostic biomarker testing for major psychiatric disorders after undergoing chorionic villus sampling. After receiving the results, these parents may decide to terminate the pregnancy if the child is at risk for a psychiatric abnormality. Couples undergoing *in vitro *fertilization already have the opportunity to select against undesirable genetic conditions by means of a pre-implantation genetic diagnosis. It is only a matter of time before psychiatric biomarkers are added to the list of conditions routinely screened for prenatally.

### Ill-treatment

There is also the potential for misusing biomarkers in a new wave of eugenics or sterilization campaigns. There is a long history of the misuse of genetic information by many governments to discriminate against those perceived as genetically unfit and to restrict their reproductive decisions. In the United States, a program of compulsory sterilization of mentally ill individuals began in 1897 with the passage of legislation in Michigan. However, the movement did not gain momentum until 1927 when the Supreme Court legitimized the forceful sterilization of patients at a Virginia home for the mentally retarded in *Beck v. Bell *[38]. Over the next 15 years, the number of sterilizations steadily increased until the case of *Skinner v. Oklahoma *complicated the legal situation [39]. This case held that compulsory sterilization could not be imposed as a punishment for a crime. Criminal sterilization laws were designed to target "criminality," believed by some at the time to be a hereditary trait. Such programs were eventually abandoned after World War II because of the association between the eugenics movement and the Nazis. During this period though, more than 65,000 individuals were sterilized in 33 states [40].

Such practices are not simply relegated to the pages of history. Current laws in China authorize the sterilization of individuals who are genetic carriers of serious medical disorders, including mental illnesses [41]. The Maternal and Infant Healthcare Law of 1995 requires couples to undergo a pre-marital medical examination. If the results of this examination reveal a "genetic disease of a serious nature which is considered to be inappropriate for child-bearing," the couple must take "long-term contraceptive measures" or undergo a sterilization procedure before they can get married [42].

## Conclusion

The eventual use of biomarkers for psychiatric diagnosis will need to be implemented with caution and with full awareness of the costs involved. Compared to current measures of disease diagnosis, such as behavioral observation or questionnaires, the use of biomarkers is a more labor-intensive approach and requires a higher level of expertise.

The long-term impact that results may have on future life choices for the individual and family members must also be investigated as genetic testing could potentially introduce misleading labels and limit an individual's opportunities. One needs to closely weigh the costs against the benefits because currently there are no reliable biomarkers that can consistently predict mental illness.

In the rush toward developing etiological screening tools, it must be remembered that the patient is at the heart of the medical profession, not their DNA. Any new diagnostic tools should confer a significant benefit to patients and not promote confusion, discrimination, or stigma.

## Competing interests

The authors declare that they have no competing interests.

## Authors' contributions

All authors participated in the preparation of the manuscript, and read and approved the final manuscript.
